# Implementing an Environmental Contaminants Deliberation Module in General Chemistry

**DOI:** 10.1021/acs.jchemed.4c00273

**Published:** 2024-07-11

**Authors:** Sara A. Mehltretter Drury, Katherine R. Knobloch, Pamela Conners, Amanda Nienow, Chris Anderson, Sidra Aghababian, Jessica Imholte, Laura M. Wysocki

**Affiliations:** † Department of Rhetoric, Wabash College, 301 W. Wabash Ave., Crawfordsville, Indiana 47933, United States; ‡ Department of Communication Studies, Colorado State University, Fort Collins, Colorado 80523, United States; § Department of Communication Studies, Gustavus Adolphus College, 800 W. College Ave., St. Peter, Minnesota 56082, United States; ∥ Department of Chemistry, Gustavus Adolphus College, 800 W. College Ave., St. Peter, Minnesota 56082, United States; ⊥ Department of Rhetoric, Wabash College, 301 W. Wabash Ave., Crawfordsville, Indiana 47933, United States; # Department of Political Science, Colorado State University, Fort Collins, Colorado 80523, United States; ■ Department of Chemistry, Gustavus Adolphus College, 800 W. College Ave., St. Peter, Minnesota 56082, United States; ○ Department of Chemistry, Wabash College, 301 W. Wabash Ave., Crawfordsville, Indiana 47933, United States

**Keywords:** First-Year Undergraduate/General, Environmental Chemistry, Interdisciplinary/Multidisciplinary, Public Understanding/Outreach, Communication/Writing, Problem Solving/Decision Making, Applications of Chemistry, Student-Centered Learning

## Abstract

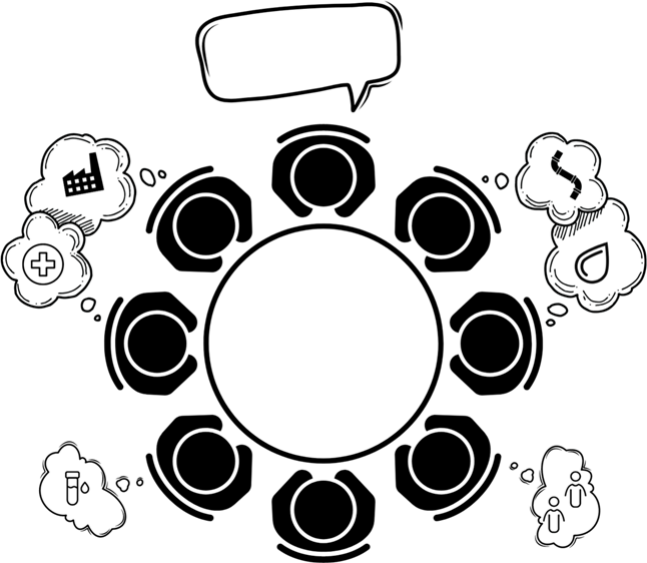

Placing chemistry in the context of complex societal issues is one way to help students see the application of fundamental ideas in the general chemistry curriculum. Here, we describe the impact of an in-class deliberation on environmental contaminants, which encourages students to consider different perspectives when addressing the issue of water and soil quality in communities. Student surveys were used to analyze the quality of the deliberation and several key factors regarding student attitudes before and after the activity. Students report a high-quality experience during the deliberation, wherein new ideas were introduced and they carefully considered different views on the issue at hand. Not only do students gain scientific knowledge about lead contamination, they also demonstrate statistically significant gains in their attitudes toward chemistry and their motivation to take action. As a complement to traditional teaching methods, this deliberation module can address key learning outcomes in systems thinking and the impact chemistry has on society.

In the first course of most chemistry curricula, General Chemistry, students are introduced to chemistry as a discipline through fundamental concepts, problem-solving, and basic laboratory techniques. To put chemistry in context, educators have looked for ways to connect classroom topics to real-world applications.^1−5^ In recent years, efforts to teach with a systems thinking approach emphasize that fundamental chemistry content can be placed within more complex systems in the real world.^6−8^ In fact, the American Chemical Society has placed an emphasis on systems thinking as a goal for approved institutions.^9^

In this context, deliberative pedagogy^10^ can be used to consider pressing issues facing society that have a technical component, or socio-scientific issues (SSI).^11−13^ Deliberation is a facilitated, small-group discussion about a social issue that invites different perspectives to be heard, with an emphasis on considering the benefits and trade-offs of different approaches to an issue. We have previously reported efforts to incorporate deliberation modules in chemistry courses for nonmajors.^14^ Deliberative pedagogy has also been used in science classrooms in a variety of formats, including in small group work in larger classrooms.^13,15,16^ Here, we extend and expand upon our prior work on deliberation modules in chemistry courses by reporting a new module, including a larger number of participants across institutions, and developing a more refined assessment strategy. This module focuses on environmental contaminants, with an emphasis on water and soil quality. It was designed for a General Chemistry course geared toward students with an interest in STEM, so we developed a conversation about the role of scientists and communication in addressing SSIs within the module. It was implemented and assessed at two small, liberal arts institutions.

Water quality and environmental contaminants encompass a significant issue facing society, and one does not need to look far to find local examples of communities dealing with contamination issues. The environmental impact of contaminants is a natural connection for green chemistry and analytical chemistry concepts to apply to real-world problems, with several examples of laboratory experiments and classroom materials.^17−21^ On a national scale, Flint, Michigan, made headlines in 2015 with the detection of very high levels of lead in their water.^22^ This high-profile case has been used to highlight issues of social justice when it comes to the communities that struggle most with environmental contaminants.^23−26^ Several of these examples happen later in a student’s coursework, but fundamental concepts often taught in general chemistry like ions, concentration, solubility, and more, can connect to water chemistry. Exposing students early in their scientific career to the role chemistry may play in a larger societal issue through deliberative pedagogy has served as motivation for our work.

We developed a 2-h deliberation module that asked students to consider stakeholders involved in the issue of water quality, allowed them to weigh different options for society to address the issue, and gave them room to consider a scientist’s role in the process. In addition to assessing the quality of the student experience, we were interested to learn whether students gained knowledge related to water quality; whether the deliberation affected students’ scientific attitudes and habits; and whether students felt motivation to take action about the issue after participating in the deliberation.

## Method

This deliberation activity was implemented at two small, residential liberal arts colleges in the Midwest in the fall semesters of 2021 and 2022, as well as spring 2023. Over the course of these 3 semesters, 300 students were enrolled and 279 completed both the preactivity and postactivity surveys associated with this project. Both institutions have a one-semester general chemistry course, with the large majority of students enrolled having a STEM interest (chemistry, biochemistry, biology, and/or prehealth). They do not share a common syllabus and the deliberation module was not synchronized, with implementation in mid to late semester. While specific classroom content may differ, connections could be made with a variety of topics, including ions, concentration, solubility, or other fundamental topics. Students in the course received a grade based on completion of the predeliberation work and participation in the deliberation. In addition, both institutions have an established cocurricular program that focuses on deliberation.^27,28^ Facilitators for deliberation came from the cocurricular program, were teaching assistants for the associated course, or were senior chemistry majors. Those who served as a facilitator for the first time were trained using resources appropriate for basic facilitation.^29,30^ One of the institutions has an all-male student body (*N* = 83) and the other is coeducational (*N* = 196). While students may have encountered deliberation in a cocurricular activity on campus, this was the first encounter with deliberation in a science course for many participants. For further demographics related to the participants in the activity, see the Supporting Information. This research was approved by the Institutional Review Board of Wabash College (IRB No. 1911181).

### The Deliberation Module

With a focus on water quality and environmental contaminants, students prepared for the deliberation by reading several articles. First, all participants read an article about Flint, MI, to help them understand the scope of the problem and some of what the community has dealt with in the years since Flint changed its water source.^31^ Second, all participants read an article about a local contamination issue. For Wabash College, this was the presence of PCE and TCE in a town outside Indianapolis.^32^ For Gustavus Adolphus College, this was PFAS contamination resulting from proximity to the 3M company.^33,34^ Finally, each student was assigned one of five scientist perspectives to read related to lead, which included pipes engineer,^35^ water chemist,^36,37^ public health expert,^38,39^ analytical research chemist,^40^ and pediatrician.^41^ Because this was an introductory course, we hoped this would allow students to visualize what a scientist in each career might do and, during the deliberation, it might enrich the conversation with exposure to scientific perspectives on the problem. Each of these readings had reading guides developed by two of the coauthors (L.M.W. and A.N., available in the Supporting Information), to aid the students in preparation; the guides were not assessed for a grade.

The deliberation took place in-person, in small groups of 6–10 students, with one trained facilitator per group. Participants were given a deliberation guide that laid out three different approaches to the issue (available in the Supporting Information). The facilitator’s role was to guide the conversation, using a facilitation guide (available in Supporting Information) that identified central questions to be discussed. The facilitator was a peer, not enrolled in the class and often not trained in chemistry, who maintained an impartial position on the issue. Their role was to pose questions of participants to encourage all voices to be heard and treated with respect. Over the course of 2 h, typically a laboratory period, the deliberation had three main stages. First, participants were asked to consider stakeholders from different roles in society, including nonscientist, nonchemistry experts, and community focused perspectives. Next, participants carefully considered the benefits and drawbacks of three different approaches to the issue of water quality: enacting harsher penalties and regulations, incentivizing positive behavior, and investing in our society to address and prevent incidents. After a short break, but still as a part of the second stage of the deliberation, participants were also asked to collectively prioritize actions and discuss the trade-offs of their chosen next steps. Finally, in the third stage, participants discussed science and public policy, including how scientists can better communicate with the public and be involved in decision-making for public issues.

### Assessment

To evaluate the deliberation and student outcomes, participants were asked to complete an online survey in the week leading up to the deliberation and another survey within 1 week after the deliberation took place. In addition to demographic information, the pre- and postsurveys asked Likert-scale questions that addressed a wide range of outcomes, including the students’ experience during the deliberation, students’ attitudes toward chemistry, their faith in the public and scientists to engage in socio-scientific decision making, and their civic attitudes and behaviors. The survey included statements about the chemistry of lead, which was a contaminant that every participant read about in the framing documents, to assess knowledge gains based on the activity. Finally, students were asked open-ended questions about their experiences deliberating and about the ways that the conversation impacted their perspective on environmental contaminants. For a complete list of items utilized in this analysis, see the Supporting Information.

## Results

Before looking into the learning outcomes of the deliberation module for students, we were first interested in their perception of the quality of the activity itself. While students in these courses are frequently engaged in active learning activities and group work in the classroom setting, facilitated deliberation is not a common experience in their science courses. Indeed, students rated their experience highly in two categories: analytic quality and democratic quality. Analytic quality was assessed with questions relating to whether they felt they learned new information and considered competing arguments and perspectives on the issue on a scale from “Definitely Not” (1) to Definitely Yes” (5) (3 items, α = 0.675 M = 4.280). Democratic quality was assessed with a scale that included questions that asked whether participants felt respected, considered others views, had trouble understanding the conversation (reverse coded), or felt pressure to agree with others (reverse coded) on a scale from “Never” (1) to “Almost Always” (5) (5 items, α = 0.675, M = 4.317).^42^ On both measures, students rated the quality highly, with average response landing between the two highest points on the scale.

Given that this activity replaced a traditional lab activity, we were also interested to know whether students learned anything related to chemistry through deliberation. To assess knowledge gains, we asked students to respond to ten statements about lead and water chemistry in both the preactivity and postactivity surveys. Students could respond on a 5-point scale that reflected confidence and correctness, such as, “I know this is correct,” “I think this is correct,” “I don’t know,” “I think this is incorrect,” and “I know this is incorrect.” This item was then recoded into a dummy variable of correct (1) or incorrect (0), with “Don’t know” coded as incorrect. The accuracy of students’ responses increased significantly after the deliberation activity, with an increase from a total average score of 6.194 (*sd* = 1.843) in the presurvey responses to a total average score of 7.040 (*sd* = 2.008) in the postsurvey responses, *t*_(277)_ = −7.137, *p* < 0.001. Though students were assigned readings prior to class, it appears that the conversation had an impact independent of the assigned readings. There was no significant correlation between the knowledge gains and the amount of readings that students said they had completed, on a scale of “did not read” (1) to “read everything carefully” (4). Moreover, an ANOVA found no significant difference in knowledge gains among students who were assigned to read different perspectives as part of their assigned readings.

In addition to gains in content knowledge, we were interested in learning about student attitudes toward science after considering a complex SSI. Several measures can be seen in Table 1. When asked about their enthusiasm about and interest in, confidence doing, and understanding of chemistry, there was a significant increase in student attitudes after the activity. In addition, there was a significant increase in students’ self-confidence in their ability to make public decisions about science (internal scientific efficacy) and in their belief that the public can influence government decisions about science (external scientific efficacy).^43^

**Table 1:** Changes to Attitudes Toward Chemistry^a^

Measure	Cronbach’s α	Pre Test *M* (sd)	Post Test *M* (sd)	*t*^b^
Interest & Enthusiasm for Chemistry	0.879	3.216 (1.038)	3.3378 (1.000)	–3.141*
Confidence in Chemistry Abilities	0.878	3.278 (0.839)	3.416 (0.815)	–3.753*
Understanding of Chemistry	0.646	3.259 (0.685)	3.583 (0.689)	–6.687*
Internal Scientific Efficacy	0.676	3.127 (0.698)	3.531 (0.655)	–10.449*
External Scientific Efficacy	0.590	3.001 (0.667)	3.139 (0.686)	–3.340*

a All scales include 3 items using 5 point scales, with 1 representing negative attitudes toward chemistry and 5 representing positive attitudes toward chemistry; alphas are derived from presurvey responses; all *t* tests had 278 degrees of freedom.

b Asterisk (*) indicates *p* < 0.001.

With the chosen SSI focus of environmental contaminants, we wanted to know whether the deliberation impacted student opinion on the issue. The results of the survey show that almost 40% of respondents reported that their views on the issue of environmental contaminants changed at least somewhat. In addition, almost three-quarters of the students who participated reported being more motivated to take action on issues related to environmental contaminants after their deliberative experience. See Table 2 for complete results.

**Table 2:** Changes to Positions and Motivations to Act

Changes to Opinions about Contaminants			Changes to Motivation to Act		
	*N*	%		*N*	%
My views are entirely the same	27	9.7	Much less motivated	1	0.4
My views are mostly the same	145	52.0	Somewhat less motivated	2	0.7
My views changed somewhat	90	32.3	Feel the same as before	72	25.8
My views changed a great deal	15	5.4	Somewhat more motivated	157	56.3
My views changed entirely	2	0.7	Much more motivated	47	16.8

## Discussion

The results related to the analytic and democratic quality of the activity demonstrate that students felt the conversation was informative and inclusive and serve as evidence of good implementation in the classroom setting. Learning outcomes for deliberative pedagogy include collaboration, understanding the complexity of public policy issues, awareness of stakeholders and relationships, and empathy.^44^ Students’ assessment of a high-quality analytic conversation reflects their learning about the issue from a range of perspectives, the discussion’s consideration of a variety of arguments, and understanding different perspectives on the issue. Additionally, the quality of democratic conversation demonstrates that student participants felt they had space to express their views, listen and consider the views of others, and promote a respectful space for deep conversation.

Beyond deliberative pedagogy outcomes, we wanted to explore the ability of this activity to help students learn basic chemistry concepts. We recognize the rigidity of many general chemistry course schedules and we hoped that substituting a traditional lab activity for this collaborative and interdisciplinary conversation would still move students forward in their knowledge gains. The statements we used as a basis for our survey questions were related to the readings students did in preparation for the deliberation and were rooted in the chemistry of lead, water chemistry, and the effect of lead on human health. While we do not have a direct comparison to other forms of pedagogy (i.e., a lecture or laboratory experiment approach to the material), we were pleased to see the significant improvement in correct responses to the statements, especially given that the in-person deliberation had an effect independent of the preactivity reading assignment. In line with the systems thinking approach, this serves as evidence that students can learn fundamental concepts as they investigate an interdisciplinary social issue.

Particularly important is the significant increase in participants’ positive attitudes toward science. While we hope the deliberation helps students understand the roles that scientists can play in addressing SSI’s, it is also understandable that complex issues without a simple solution can be intimidating and frustrating. For students in general chemistry, which can be seen as a gateway course for STEM majors, a significant increase in enthusiasm and confidence related to chemistry is a positive outcome. Keeping students engaged with chemistry, especially if that includes working with real-world complex ideas in chemistry, serves as an encouraging early experience for students and, in some cases, as an example they can relate to later in their careers. Beyond this attitude toward chemistry, students showed significant gains in areas that emphasize the relationship between science and community issues–both internal and external scientific efficacy. The activity prompted changes in students’ perception of the issue and their own civic agency. For early career science students, the high motivation to act after the deliberation connects an understanding of their developing role as ethical scientists *and* active, engaged citizens. If students with a scientific expertise become empowered to contribute to public decision-making and feel as if their participation makes a difference, it could positively affect their current and future communities.

Finally, we looked at the urgency surrounding the SSI itself. After this in-depth examination of environmental contaminants, we hoped that students would be more aware of the water they drink and what is in their environment. It stands to reason that students may have carefully considered these issues before participating in the deliberation based on their lived experience. However, for the majority of the participants, their survey responses suggested a change in their views or an increase in their motivation to act on the issue. While these numbers are self-reported, increased awareness of the issue, careful consideration of how society may deal with it, and the potential to take steps to improve the issue are all positive outcomes.

## Conclusion

Overall, the development and implementation of a deliberation module based on environmental contaminants demonstrated positive learning outcomes related to deliberative pedagogy, chemistry knowledge, attitudes toward chemistry, and motivation to act on the issue. This intervention is relatively short, low-cost, and can be implemented in a variety of introductory chemistry course settings, leading to high impacts in student learning. This work represents implementation at two institutions and did not require synchronization of the course schedules. While both institutions described here have a deliberation program for undergraduates housed outside the chemistry department, future research will involve the development of a virtual training session for facilitators that do not have previous experience in the role and the implementation of this module at a different institution without on-site deliberation resources. These deliberations were done in-person, but one could imagine adapting the module for a virtual environment, keeping in mind best practices for online teaching.^45−47^ Additionally, the resources for this module, including a facilitator guide and participant guide, are available for implementation in the Supporting Information.

## Supporting Information Available

Facilitation Guide; Contaminants Deliberation Placemat Guide; Reading Guides; Demographic Data Table; Survey Measures (PDF)
