# Supplementary material for: Implementing an Environmental Contaminants Deliberation Module in General Chemistry
Source: J Chem Educ. 2024 Jul 11;101(8):3564–9. doi: 10.1021/acs.jchemed.4c00273 (PMC11328133; doi:10.1021/acs.jchemed.4c00273)
Supplement: Supplementary file 1 — ed4c00273_si_001.pdf [file ed4c00273_si_001.pdf]

Supporting Information

Implementing an Environmental Contaminants Deliberation Module in General Chemistry

Sara A. Mehltretter Drury[1], Katherine R. Knobloch[2], Pamela Conners[3], Amanda Nienow[4], Chris Anderson[1], Sidra Aghababian[2], Jessica Imholte[4], Laura M. Wysocki[5]*

[1]Department of Rhetoric, Wabash College, Crawfordsville, IN 47933, USA

[2]Department of Communication Studies, Colorado State University, Fort Collins, CO 80523, USA

[3]Department of Communication Studies, Gustavus Adolphus College, Saint Peter, MN 56082, USA

[4]Department of Chemistry, Gustavus Adolphus College, Saint Peter, MN 56082, USA

[5]Department of Chemistry, Wabash College, Crawfordsville, IN 47933, USA

*To whom correspondence should be addressed: wysockil@wabash.edu

Table of Contents

Demographic Data Table S2

Survey Measures S3–S7

Reading Guides S8–S13

Contaminants Deliberation Facilitation Guide S14–S21

Contaminants Deliberation Placemat Guide S22

Demographic Data

Table S1. Demographic Data, Deliberation Participants, n=298

Class Year		Other Characteristics	
Freshman	65.4%	Pell-eligible	18.8%
Sophomore	25.8%	First Generation	11.0%
Junior	6.7%		
Senior	2.0%		

Table S2. Demographic Data, Survey Respondents, n=279

Gender		Race	
Man	155	African American	6
Woman	113	Asian American	11
Other	7	Hispanic or Latinx	9
		White, Non-Hispanic	222
		Other	5
		More than one race	17
**Age**		**Science Major**	
18	106	Science Major	253
19	111	Non-Science Major	21
20	43	**Chemistry Major**	
21	8	Chemistry Major	67
22	6	Non-Chemistry Major	207
24	1		
69	1		
**Party Affiliation**		**Ideology**	
Democrat	101	Liberal	104
Republican	80	Moderate	80
Independent/No Party	25	Conservative	91
Don't Know/Decline	73		

Survey Measures

Analytic Quality (from Knobloch, Gastil, Reedy, & Walsh, 2013)
Items:

1. Do you believe that you learned enough in the deliberation activities to have an informed opinion about the issues discussed?
2. Did this deliberation(s) encourage you to weigh the most important arguments and issues relating to the deliberation topic?
3. Did the conversation help you understand different people's perspectives on this issue?

Response Options:

1. Definitely Not
2. Probably Not
3. Unsure
4. Probably Yes
5. Definitely Yes

Democratic Quality (from Knobloch, Gastil, Reedy, & Walsh, 2013)
Items:

1. How often during the deliberation did you feel you had sufficient opportunity to express your views?
2. When others expressed views different from your own during the deliberations, how often did you consider carefully what they had to say?
3. How often during the deliberation do you feel that other participants treated you with respect?
4. How often during the deliberation did you have trouble understanding or following the discussion? (reverse coded)
5. How often during the deliberation did you feel pressured to agree with something that you weren't sure about? (reverse coded)

Response Options:

1. Never
2. Rarely
3. Occasionally
4. Often
5. Almost Always

Knowledge Items
Items:

1. Oxidation of lead pipes increases the metal in the solution.
2. All lead compounds dissolve in water.

3. Boiling water reduces metal contaminants.
4. Sitting water generally has more contaminants than flowing water.
5. Children have a relatively higher risk of health issues associated with lead than adults.
6. Lead is still used widely in commercial products.
7. Lead accumulates in the body over time.
8. Increased lead exposure can lead to neurological issues.
9. Lead poisoning always results from a recent high exposure of lead.
10. Dangerous exposure to lead is primarily through the skin.

Response Options:

1. I know it is correct
2. I think it is correct
3. I am unsure if it is correct or incorrect
4. I think it is incorrect
5. I know it is incorrect

Interest in and Enthusiasm for Chemistry

Item: In this section, we'd like to ask you some questions about your knowledge of chemistry. Please reply to the following questions with how much you presently understand the topic.

1. Enthusiastic about chemistry
2. Interested in discussing chemistry with friends or family
3. Interested in taking additional classes in this subject
4. Planning to take additional classes in this subject

Response Options

1. Not at all
2. A little
3. Somewhat
4. A lot
5. A great deal

Confidence in Chemistry Abilities

Item: In this section, we'd like to ask you some questions about your knowledge of chemistry. Please reply to the following questions with how much you presently understand the topic.

1. Confident that I understand chemistry
2. Confident that I can do chemistry

3. Comfortable working with complex ideas in chemistry

Response Options
1. Not at all
2. A little
3. Somewhat
4. A lot
5. A great deal

Understanding of Chemistry
Item: In this section, we'd like to ask you some questions about your knowledge of chemistry. Please reply to the following questions with how much you presently understand the topic.
1. Chemical principles relating to water and environmental quality
2. How ideas we explore in chemistry relate to ideas I have encountered in other science classes
3. How studying chemistry helps people address real world issues

Response Options
1. Not at all
2. A little
3. Somewhat
4. A lot
5. A great deal

Internal Scientific Efficacy (adapted from Morrell, 2003)
Item:
1. I feel confident in my ability to make decisions about community issues that deal with science.
2. I think that I am more informed about the ways that science influences political decisions than most people.
3. I consider myself well qualified to take part in public decision making related to scientific issues.
4. People like me don't have any say in government decisions that relate to science.
5. Public officials listen to people like me when making decisions about scientific issues.
6. There are many ways for members of the public to influence government decisions related to science.

Response Options
1. Strongly Disagree
2. Disagree
3. Neutral
4. Agree
5. Strongly Agree

External Scientific Efficacy (adapted from Morrell, 2003)
Item:
1. People like me don't have any say in government decisions that relate to science.
2. Public officials listen to people like me when making decisions about scientific issues.
3. There are many ways for members of the public to influence government decisions related to science.

Response Options
1. Strongly Disagree
2. Disagree
3. Neutral
4. Agree
5. Strongly Agree

Opinion Change
Item: How did the class deliberation shape your opinion on the issue of environmental contaminants?

Response Options
1. My views are entirely the same as before
2. My views are mostly the same as before
3. My views changed somewhat
4. My views changed a great deal
5. My views changed entirely

Motivation to Act
Item: After our discussion, how motivated are you to take action related to environmental contaminants?

Response Options
1. I am much less motivated to act than before

2. I feel somewhat less motivated to act than before
3. I feel the same as before
4. I feel somewhat more motivated to act than before
5. I feel much more motivated to act than before

Reading Assignment Completion
Item: How much of the assigned readings did you complete prior to class? This question, like all others on this survey, will be used solely for research purposes, and your instructor will not have access to your answer.

Response Options
1. I read everything carefully.
2. I read most of it.
3. I skimmed the readings.
4. I did not do the assigned readings.

Reading Guide
The Flint water crisis: A loss of trust (CBS News)
https://www.cbsnews.com/news/the-flint-water-crisis-a-loss-of-trust/

As you read or watch the video, consider and respond to the following questions:

1. What were some areas of concern highlighted by experts in this news story?

2. Why did the lead levels in Flint's water suddenly rise?

3. What are some ways the government and the Flint community has responded to finding lead in their water?

Reading Guide
Participant 1, 6 – Pipes Engineering

Your reading is an excerpt of a report from the Water Research Foundation and the American Water Works Association. As you read, consider and respond to the following questions:

1. What are common sources of lead exposure?

2. Explain how lead ends up in the water.

3. Chemically, what is different between soluble and insoluble lead in the water?

4. How can water characteristics be altered to minimize lead in the drinking water? Describe several approaches and the benefits or challenges associated with them.

Reading Guide
Participant 2, 7 – Water Engineering

Your reading Is “The History of Drinking Water Treatment” and “The Chlorine Dilemma.” As you read, consider and respond to the following questions:

1. What are some of the factors that led to the Safe Drinking Water Act of 1974?

2. What are the benefits and the tradeoffs of treating water with chlorine or related chlorine compounds?

3. What are different water treatments that we could decide to use, and what are their tradeoffs?

Reading Guide
Participant 3, 8 – Public Health

Your reading includes two case studies in controlling lead exposure: one from Providence, RI, and one from Smeltertown. As you read, consider and respond to the following questions:

1. Briefly summarize why the community in Providence, RI, felt an urgency about lead exposure.

2. Describe the strategies that the Urban Environmental Initiative (UEI) used in Providence to prevent lead exposure.

3. Who were the stakeholders in conversation about Smeltertown's lead exposure? What tensions existed within this community?

4. What steps were taken to deal with lead exposure in Smeltertown? Briefly explain their consequences.

Reading Guide
Participant 4, 9 – Research Chemist

Your reading comes from the World Health Organization (WHO). As you read, consider and respond to the following questions:

1. Briefly explain why it is important to be able to accurately measure lead in a sample. What are some of the challenges in accurate measurement of lead levels?

2. Briefly summarize methods used to detect lead in water or blood.

3. What are good laboratory practices for collecting and handling samples?

4. How do researchers choose which method of lead detection to use for a given sample?

Reading Guide
Participant 5 – Pediatrician

Your reading comes Your reading comes from the Agency for Toxic Substances and Disease Registry of the Centers for Disease Control, and is a subset of a Continuing Medical Education (CME) course that physicians can take to maintain their licensure. There is a lot of jargon in this article, so do your best to understand the main points. As you read, consider and respond to the following questions:

1. How does lead exposure generally occur?

2. Why are children particularly at risk of lead poisoning?

3. Where does lead exist in the body after exposure?

4. What are the physiological effects of lead poisoning?

5. How is lead poisoning diagnosed?

Environmental Contaminants Deliberation Facilitation Guide

Time: 2-2.25 hours

This guide was created with contributions from Sara A. Mehltretter Drury (Wabash College), Laura M. Wysocki (Wabash College), Arthur Equihua (Wabash College '20), Hayley Blonsley (Wabash College), Amanda Nienow (Gustavus Adolphus College), and Pamela Conners (Gustavus Adolphus College).

Activity Description:

You will participate in a deliberation about environmental contamination, with a focus on water quality. Contaminants in our soil and water can have significant, dangerous impacts on our environment and our overall health. Recently in the United States, it seems like issues of contaminants in the soil and water most impact those who are already struggling to make ends meet. In this lab, you will deliberate about how to counter effects and prevent future incidents.

A deliberation is a rational process through which participants consider different perspectives in order to collaboratively arrive at a decision. Your conversation will be guided by a trained facilitator, who will ask you to examine the problem of water and soil contaminants carefully and weigh the benefits and tradeoffs of different approaches for addressing it.

Preparation Work for students and facilitators

All students:

- Watch video: https://www.cbsnews.com/news/the-flint-water-crisis-a-loss-of-trust/
- Read news local news stories:

  Indiana Selections:
  (1) https://www.indystar.com/story/news/environment/2018/11/05/franklin-indiana-sewers-have-cancer-causing-chemicals-test-shows/1857362002/
  Minnesota Selections:
  (2) https://www.mprnews.org/story/2021/03/18/forever-chemicals-found-in-groundwater-at-dozens-of-minn-landfills
  (3) https://www.mprnews.org/story/2020/09/10/state-offers-options-to-keep-st-paul-suburban-water-free-of-3m-chemicals

- Read stakeholder perspective as assigned.
- Answer questions on reading guide ahead of deliberation.

Ideally, deliberation groups should be a minimum of 5 students and a maximum of 10.
One facilitator is needed per group.

Student Reading Assignments

Participant 1, 6 (Pipes Engineering)	Your reading comes from the Water Research Foundation ("Controlling Lead in Drinking Water)".
Participant 2, 7 (Water Engineering)	Your reading are essays from the Environmental Protection Agency ("The History of Drinking Water Treatment") and *Science* ("The Chlorine Dilemma").

Participant 3, 8 (Public Health)	Your reading includes two case studies in controlling lead exposure: one from Providence, RI (EPA) and one from Smeltertown (Romero, 1984).
Participant 4, 9 (Analytical Research Chemist)	Your reading comes from the World Health Organization (WHO).
Participant 5, 10 (Pediatrician)	Your reading comes from the Agency for Toxic Substances and Disease Registry of the Centers for Disease Control ("Initial Check"), and is a subset of a Continuing Medical Education (CME) course that physicians can take to maintain their licensure.

Facilitation Plan

Agenda (2 hours):

1. Opening Framing (5 minutes)
2. Introductions in Groups (5 minutes)
3. Stage 1: Gaining Perspective of the Different Roles in Society (20 minutes)
4. Stage 2: Deliberation with group (45 minutes)
5. *Break (5 minutes)*
6. Stage 2 continued: Deliberation with group (20 minutes)
7. Stage 3: Science and Civics (20 minutes)
8. Conclusion (5 minutes)

1. Opening Framing of the Deliberation.
***As class begins*: Instructors should introduce the deliberation activity; sample remarks are presented here.** (5 min)

Instructor:
This week, our class will be investigating the issue of environmental contaminants from a deliberative perspective. Public deliberation is a group communication and critical thinking process that enables us to consider public issues from many perspectives, weigh options, and then decide what should be done. Deliberations bring together facts and experiences from many people, including scientists, government officials, members of the public, and more. Deliberation encourages the *slow* consideration of a problem so that multiple perspectives can be explored through different actions, each with benefits and tradeoffs.

Today, you will be taking part in a deliberation. Your group will engage a variety of public experiences of the issue of contaminants in the water and soil, consider benefits and tradeoffs of actions, and ultimately, decide what should be done.

We'll turn it over to your small group facilitators so they can begin this exercise.

[*Instructor and may leave the room or sit off to the side. Instructor should not participate in or interrupt the deliberation. Students may feel less likely to deliberate openly if the instructor is*

"listening" or contributing. The goal is that students are able to discuss in a peer-to-peer setting.]

2. Introductions in Groups (10 min)

[Facilitators should introduce themselves]

Hello, my name is ____________, and I will be the facilitator today. My role is to guide the conversation in an impartial, neutral way.

There are a few guidelines that we would like to lay out that will make this deliberation successful.

- First, we encourage everyone to participate, and no one or two individuals should dominate.
- Second, listen to others. Listening is as important as speaking in a deliberation.
- Third, keep an open mind to new ideas. Avoid conclusions until we have discussed all the options.
- Fourth, disagree with curiosity; the purpose of deliberations is to learn about the issue and others' perspectives.
- Finally, please silence your cell phones.

[note to facilitators: please practice this ahead of time so that you can deliver/read it with energy and enthusiasm]

Today we will take time to consider the public problem around environmental contaminants in our water and soil. The issue of contaminants is a complex scientific issue that involves various types of scientific expertise and knowledge. It also is a complicated **public** issue, which involves many different stakeholders.

In fact, this issue is so complicated, we might call it a **wicked problem**. Wicked problems are public issues with many stakeholders that are hard to understand and challenging to address through single actions. Furthermore, any action step or solution to a wicked problem has tradeoffs that have to be managed—no action is perfect, and so part of the work of **deliberation** is to weigh the **benefits** and **drawbacks** of different actions.

So today, we'll explore a variety of potential approaches, and spend time working through different aspects of the issue. Ultimately, we want you to use this experience to decide how best to manage the problem of contaminants in the environment.

In the first stage of the deliberation, you'll think about different perspectives on this problem, using the readings you did before today's lab period to inform our understanding of the issue.

In the second stage of the deliberation, you will then consider three potential approaches to addressing and managing this problem. After weighing different options you will then decide what should be done.

In the final stage, you'll consider the role of scientists in addressing scientific public issues and in engaging the public.

Before we begin, let's introduce ourselves by sharing our names.

3. Stage 1: Gaining Perspective of the Different Roles in Society (20 minutes)

Our first task today is to think about this issue as a public, wicked problem. You all watched a video on Flint, Michigan, and read the news article about contamination in ____________________ ____________________[*insert city/state from readings*]. These news sources talk about the issue from the public perspective. You also had a chance to read a perspective of a scientific stakeholder on environmental contaminants. We'll focus our conversation for the next 20 minutes on the different stakeholders around this issue.

Let's talk more about what we learned about the issue.

Questions:

- Let's focus first on public perspectives of the issue, or non-scientist perspectives. Based on the news reports who is concerned about this issue? Who does this issue impact?
  - *Have them get specific—should produce different professions and community groups. Should include community-perspectives as well as experts.*
  - For each stakeholder mentioned, what information do they bring to the issue? What should we be aware of?
  - For each stakeholder mentioned, what are their concerns about the issue?
  - Are there stakeholders in the public missing from these news reports?

- Now let's focus on the expert perspectives you read.
  - First, let's go around and share a brief summary of what you read. What does your perspective bring to the issue?
  - Some of you read the same perspectives, so if you have someone who read what you read, please speak up immediately after them.
  - *[Once everyone has shared]* What stakes do these experts have in the problem of contaminants?

- As you think about the different stakes amongst members of the public, government, and scientific experts, are there any conflicts or tensions amongst the concerns we've discussed?
- Are there questions that remain about the facts or circumstances of this issue? By this we mean what the problem is, not what should be done, because we'll be deliberating what should be done
  - *[Facilitator: you might list the questions and then ask your if anyone in your group can answer from their knowledge or perspective reading, or if the information is not known for the members of the group.]*

Student Perspective Reading Assignments (for facilitators' reference)

Participant 1, 6 (Pipes Engineering)
Participant 2, 7 (Water Engineering)
Participant 3, 8 (Public Health)
Participant 4, 9 (Research Chemist)
Participant 5, 10 (Pediatrician)

4. Stage 2: Deliberation with group (60 minutes)
Facilitator: Now that we've thought more about the information and perspectives on the issue, let's turn to deliberating about different ways of approaching and addressing the problem.

Many communities are grappling with the issue of contaminants in the environment, including Flint, Michigan; [*insert city/state from readings*]. Today, we're going to deliberate how we can best protect against future issues with contaminants in our environment. To help our discussion, we will address this problem by considering three options, and then we will decide which are the best actions for the future.

Does everyone have a copy of the guide that they can look at? *[Facilitators can give extra guides to those who may not have a paper version of the "Placemat" guide].*

As you see on this chart, each option suggests a different focal approach to the problems of contaminants. The **options are not entirely exclusive; for example, multiple actions from one or two approaches could be selected.** However, we know that in addressing public problems, there are limited resources of time and personnel. So we have to make choices **about what is most important.** Today, we will discuss these options to make a better decision about which are the best possible actions going forward.

We'll spend about 15 minutes on each option, to highlight possible actions, benefits, and tradeoffs. Then, we'll spend time talking about what should be done in the future.

Option One: Enact harsher penalties and regulations for those who do not protect the public (15 min)

Facilitator: Could one of you please read the first option?
Participant Reads: *This option suggests that companies and governments do not always respond ethically to issues of contaminants in the soil. Harsher regulations and penalties may decrease future incidents.*

Facilitator: Thank you.

- What are some initial thoughts to this option?
- Who would benefit from this option?
- Are there any actions you think would be a good response?
- In addition the actions listed, what else might be done under this approach?
- What are potential drawbacks or concerns about this option?

    - One possible drawback is that there are already laws and regulations in place, but incidents continue to happen. Do you believe harsher penalties make an impact?
    - Will penalties foster greater accountability in companies?
- Can any of the drawbacks be addressed or managed to increase the benefit?
- What seems to be <u>most important</u> reason for pursuing this approach? *[Or, why might someone be in favor of pursuing this approach]*

*[At the end, the facilitator should give a **brief** (20-30 second) summary of what the group has discussed, reviewing benefits, tradeoffs, and any tensions you've heard, before moving to the next approach]*

Option Two: Incentivize positive behavior (15 min)

Facilitator: Could one of you please read the second option?
Participant Reads: *This option assumes that contaminants will continue to cause problems for U.S. communities, and places a funding priority on prevention, testing, and education programs.*

Facilitator: Thank you.

- What are some initial thoughts to this option?
- Who would benefit from this option?
- Are there any actions you think would be a good response?
- In addition the actions listed, what else might be done under this approach?
- What are potential drawbacks or concerns about this option?
    - One possible drawback is that this doesn't help places like Flint and East Chicago (<u>add in communities close to you</u>) who have been impacted. How can we balance protecting future risk with helping those already suffering?
- What seems to be <u>most important</u> reason for pursuing this approach? *[Or, why might someone be in favor of pursuing this approach]*

*[At the end, the facilitator should give a **brief** (20-30 second) summary of what the group has discussed, reviewing benefits, tradeoffs, and any tensions you've heard, before moving to the next approach]*

Option Three: Invest in our society to address and prevent incidents (15 min)

Facilitator: Could one of you please read the third option?
Participant Reads: *This option assumes contaminants can be reduced or prevented, and places a priority on developing better infrastructure and training others to respond quickly and ethically to incidents.*

Facilitator: Thank you.

- What do you think about this option?

- Who would benefit from this option?
- Are there any actions you think would be a good response?
- In addition the actions listed, what else might be done under this approach?
- What are potential drawbacks or concerns about this option?
    - One drawback is that contaminant issues can be hard to predict. How do we balance that uncertainty with the cost of investing in systems?
- What seems to be <u>most important</u> reason for pursuing this approach? *[Or, why might someone be in favor of pursuing this approach]*

*[give a **brief** (20-30 second) summary of what the group has discussed, reviewing benefits, tradeoffs, and any tensions you've heard, before moving to the next approach]*

Facilitator: Thank you. We're going to take a 5 minute break. Feel free to stretch or use the restroom. Please be ready to discuss again at ____[time].

<u>**5. Break (5 min)**</u>

<u>**6. Prioritizing Actions / Decision-Making** (20 min)</u>

Facilitator: Now that we have discussed the three options, let's bring everything together and weigh our decisions.

<u>*[facilitator should give a summary of the three approaches—be careful not to offer conclusions, but rather key ideas of what the group has said what should be done]*</u>

- Now that we've talked about three different options, are there other possibilities exist for actions?
- Thinking across all the options, what actions or priorities seem <u>most</u> important to do?
    - Why are these so important to addressing this issue?
    - What are the tradeoffs of these actions or priorities? Can the tradeoff be managed, or minimized? Or, are we willing to live with the tradeoff?
- Who needs to be involved in addressing this problem?
    - [[if they point only to institutions/large entities]] Are there ways that individuals can take part in addressing this issue?
    - How can the public be involved?
    - How can science professionals take part in addressing this issue?
- How should our society proceed in the future to address this problem?

Facilitator: *[provide a brief summary of the conversation]*

<u>**7. Stage 3: Science and Civics (20 minutes)**</u>

Facilitator: Now that we've talked about this problem, let's take a step back and talk about science and public policy.

- What makes scientific wicked problems so hard to discuss?
- Do you feel like scientists and experts do a good job communicating with the public? Why or why not?

- *<u><<If they talked about education or the need for better information in their answers>></u>* You mentioned scientific communication and education. How can scientists communicate these ideas more effectively?
    - What strategies would you use, as a science student, to discuss these ideas in your communities if they faced environmental contaminants?
- *<u><<If they DID NOT talk about education or the need for better information>></u>* How might scientists contribute to addressing this problem?
    - How might scientists encourage more effective communication?
    - What strategies would you use, as a science student, to discuss these ideas in your communities if they faced environmental contaminants?

- What are positive ways that scientists engage with the public?
- What are positive ways that the public engages with science and scientists?
    - What are different public engagement strategies you have observed?
    - What has frustrated you about public engagement on science issues?

- How can the public be more involved in decision making for public issues relating to science?
- How can scientists be more involved in decision making for public issues relating to science?
    - In your view, who needs to be involved in these sorts of public issues?

- Based on our conversations in this deliberation, what will you take with you as you think about scientists and public engagement?

[Facilitator should offer a summary at the end, and thank everyone for attending]

How can we best protect against contaminants?

Contaminants in our soil and water can have significant, dangerous impacts on our environment and our overall health. Recently in the United States, it seems like issues of contaminants in the soil and water most impact those who are already struggling to make ends meet. Our question to deliberate today is how to counter effects and prevent future incidents.

OPTION ONE: Enact harsher penalties and regulations for those who do not protect the public.	OPTION TWO: Incentivize positive behavior.	OPTION THREE: Invest in our society to address and prevent incidents.
This option suggests that companies and governments do not always respond ethically to issues of contaminants in the soil. Harsher regulations and penalties may decrease future incidents.<br><br><u>Possible Actions to take in this approach:</u><br>☑ Take away business licenses for companies that don't abide by regulation<br>☑ Impose mandatory minimum penalties for government officials who are found guilty of ignoring or misleading the public about a problem<br>☑ Issue fines to businesses and individuals<br><br><u>Possible Drawbacks to this approach:</u><br>⇩ There are already regulations in place and incidents have continued to impact communities<br>⇩ Penalties after the fact do not always address the harms inflicted on communities; for example, sometimes companies are already bankrupt or closed and cannot financially assist communities	This option assumes that contaminants will continue to cause problems for U.S. communities, and places a funding priority on testing, education, and mitigation.<br><br><u>Possible Actions to take in this approach:</u><br>☑ Add tax breaks for "green" companies that help the environment and communities<br>☑ Conduct education programs for citizens, government leaders, and scientists to encourage greater awareness<br>☑ Consumers could prioritize patronizing companies who take protective steps to address contaminants<br><br><u>Possible Drawbacks to this approach:</u><br>⇩ Incentives such as tax breaks merely encourage, but don't compel change<br>⇩ This approach does not assist communities that are already severely impacted<br>⇩ It may be difficult for citizens to gauge how to use their patronage and buying power	This option assumes contaminants can be reduced or prevented, and places a priority on developing better infrastructure and training others to respond quickly and ethically to incidents.<br><br><u>Possible Actions to take in this approach:</u><br>☑ Develop and implement rapid response mechanisms, learning from failures in recent cases<br>☑ Replace at-risk infrastructure<br>☑ Provide grants to companies and governments for new, innovative environmental initiatives<br><br><u>Possible Drawbacks to this approach:</u><br>⇩ Even with strong proactive prevention, it would be challenging to be involved in every single potential case in the United States<br>⇩ Infrastructure projects can be very expensive, and not <u>all</u> old pipes result in contaminants<br>⇩ Innovation can be expensive and take time

What should be done? And who should take actions?

Developed by the Wabash Democracy and Public Discourse Initiative
Revised Fall 2022
